# Threshold effect of white blood cell count on the risk of refractory *Mycoplasma pneumoniae* pneumonia in pediatric patients: A retrospective cohort study

**DOI:** 10.1097/MD.0000000000044999

**Published:** 2025-10-03

**Authors:** Lingke Liu, Xuan Wenjie, Yana Wang, Xiaoxian Wang

**Affiliations:** a Department of Pediatrics, The Affiliated Hospital of Shaoxing University, Shaoxing, Zhejiang Province, PR China; b Department of Pediatrics, Shaoxing People’s Hospital (Shaoxing Hospital, Zhejiang University School of Medicine), Shaoxing, Zhejiang Province, PR China.

**Keywords:** pediatric pneumonia, RMPP, threshold effect, WBC

## Abstract

Refractory *Mycoplasma pneumoniae* pneumonia (RMPP) remains a major challenge in pediatric respiratory infections, with limited early predictors for disease progression. White blood cell (WBC) count is a widely used biomarker, but its relationship with RMPP risk has not been fully elucidated. This study investigates the threshold effect of WBC count on RMPP development to enhance risk stratification and early intervention strategies. A retrospective cohort study was conducted on pediatric patients diagnosed with MPP. WBC count was analyzed as both a continuous variable and stratified into quartiles to evaluate its association with RMPP risk using multivariable logistic regression. A threshold effect analysis was performed, and model selection was determined by the log-likelihood ratio test (*P* = .017). Adjustments were made for age, gender, weight, inflammatory markers (C-reactive protein, interleukin-6, γ-IFN), and metabolic indicators (lactate dehydrogenase, lactate, ALT, AST). A nonlinear relationship between WBC count and RMPP risk was identified. A threshold at WBC = 14.3 × 10^9^/L was detected, which serves as a critical diagnostic indicator. When WBC count exceeds this threshold, it strongly suggests the development of RMPP. For WBC levels below 14.3 × 10^9^/L, each 1 × 10^9^/L increase was associated with a 20% higher RMPP risk (OR = 1.2, 95% CI: 1.1–1.2, *P* < .001), indicating that moderate WBC elevations are a strong predictor of disease progression. The WBC count was associated with the development of refractory RMPP. WBC count could be used as a crucial biomarker for risk stratification and clinical decision-making in pediatric MPP, providing clinicians with a clear threshold for early intervention and close monitoring.

## 1. Introduction

*Mycoplasma pneumoniae* pneumonia (MPP) is a leading cause of community-acquired pneumonia in children, accounting for a substantial proportion of pediatric respiratory infections worldwide.^[1–5]^ While most cases of MPP are self-limiting and respond well to macrolide antibiotics, a growing number of cases exhibit persistent fever, clinical deterioration, and radiological progression despite appropriate macrolide therapy, a condition termed refractory *Mycoplasma pneumoniae* pneumonia (RMPP).^[6–10]^ The exact mechanisms underlying RMPP remain unclear, the excessive host immune responses and macrolide-resistant *M pneumoniae* strains play critical roles in disease progression.^[11]^ RMPP may be associated with heightened immune responses or bacterial resistance, thus necessitating prompt corticosteroid treatment.^[12–14]^ Early risk stratification and identification of biomarkers predictive of RMPP progression are essential to guide timely clinical interventions and improve outcomes.

The white blood cell (WBC) count is one of the most commonly used inflammatory markers in clinical practice.^[15,16]^ However, its predictive value in RMPP remains controversial, as some studies suggest that WBC count may not always correlate with disease severity due to the complex interplay between inflammation, immune regulation, and bacterial pathogenesis.^[17]^ The neutrophil-to-lymphocyte ratio, C-reactive protein (CRP), and interleukin-6 (IL-6) have been proposed as alternative markers, yet their predictive performance is inconsistent.^[18]^ Given the lack of consensus, a more detailed analysis of the relationship between WBC count and RMPP risk, particularly potential threshold effects, is warranted. Identifying a specific WBC threshold that predicts RMPP development could offer a simple yet effective tool for early risk assessment, allowing clinicians to implement targeted interventions before disease progression.

This study aims to investigate the critical threshold of WBC count in identifying RMPP in pediatric patients. By examining the relationship between WBC count and RMPP risk, we seek to provide clinicians with a clear, actionable indicator for early detection and intervention.

## 2. Materials and methods

### 2.1. Patients

**Figure 1.**
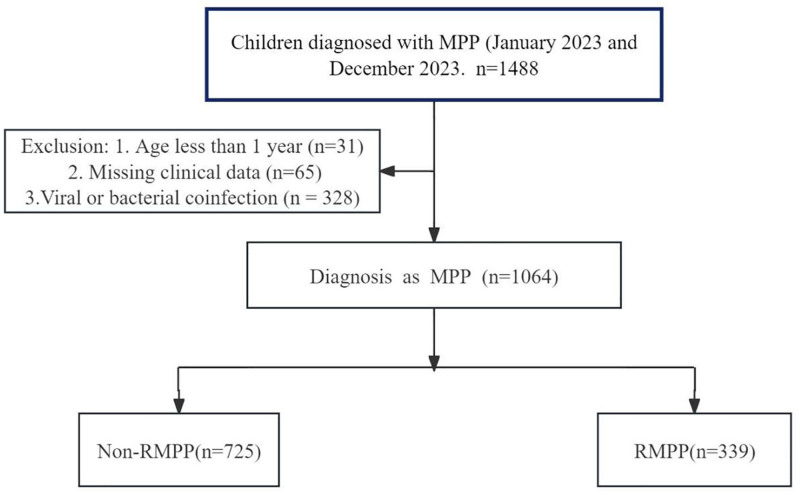
Study flowchart. A flowchart illustrating the patient selection process for the study on *Mycoplasma pneumoniae* pneumonia (MPP) and refractory *Mycoplasma pneumoniae* pneumonia (RMPP). After exclusions, 1064 cases were confirmed as MPP. These were further classified into 2 subgroups: 725 cases of non-MPP and 339 cases of RMPP, based on clinical characteristics such as persistent fever, worsening radiological findings, and response to macrolide therapy. MPP = *Mycoplasma pneumoniae* pneumonia, RMPP = refractory *Mycoplasma pneumoniae* pneumonia.

This retrospective cohort study was conducted among hospitalized pediatric patients diagnosed with MPP at The Affiliated Hospital of Zhejiang University between January 2023 and December 2023.^[19]^ Inclusion criteria consisted of patients aged 1 to 14 years who met the clinical and laboratory diagnostic criteria for MPP, including positive *M pneumoniae* IgM antibody tests, confirmation by polymerase chain reaction, and characteristic radiological findings.^[1]^ Patients with coinfections, incomplete medical records, congenital or chronic pulmonary diseases, or immunodeficiencies were excluded. Patients with confirmed coinfections, including viral (e.g., influenza, RSV, adenovirus) or bacterial pathogens (e.g., *Streptococcus pneumoniae, Haemophilus influenzae*), as determined by respiratory pathogen panel testing, were also excluded. Participants were categorized into RMPP and non-RMPP (non-RMPP) groups based on clinical progression and therapeutic response (Fig. 1).^[7]^

### 2.2. Ethical statement

This study was conducted in accordance with the ethical standards outlined in the Declaration of Helsinki and was approved by the Ethics Committee of The Affiliated Hospital of Zhejiang University (Approval No. 2023-IRB-0042-P-01). Given the retrospective nature of the study, the requirement for informed consent was waived by the ethics committee.

### 2.3. Data collection

Demographic, clinical, and laboratory data were extracted from electronic medical records. Key variables included age, gender, weight, fever duration, hospitalization duration, and inflammatory markers (CRP, erythrocyte sedimentation rate, IL-6, γ-interferon [γ-IFN], and lactate dehydrogenase [LDH]).^[18]^ The WBC count was collected at the time of hospital admission, before the initiation of corticosteroid therapy. All patients were included based on laboratory results obtained within 24 hours of admission and prior to systemic immunomodulatory treatment, ensuring consistency in baseline inflammatory status. The WBC count on admission was analyzed as a continuous variable and stratified into quartiles. Pulmonary imaging data and treatment regimens were also recorded. Patients with incomplete medical records, including missing laboratory or clinical outcome data, were excluded from the analysis to maintain dataset integrity and avoid potential bias from imputation.

### 2.4. RMPP clinical diagnostic criteria

RMPP was diagnosed according to established guidelines.^[11]^ Diagnostic criteria included persistent fever (≥38.5°C) lasting <7 days despite macrolide treatment, progressive radiological deterioration such as increased pulmonary consolidation or development of pleural effusion, significantly elevated inflammatory markers (e.g., CRP, IL-6, γ-IFN), and inadequate clinical improvement with macrolide monotherapy.^[7,17,18]^

In such cases, adjunctive corticosteroid therapy and/or a change in antibiotic regimen were considered.^[12]^ Specifically, corticosteroid therapy, such as methylprednisolone at 1 to 2 mg/kg/d, was initiated to reduce inflammation.^[20]^ If there was no clinical improvement, pulse therapy with higher doses (10–30 mg/kg/d) for 3 days was administered.^[21]^ Additionally, for patients unresponsive to macrolides, alternative antibiotics like doxycycline (4 mg/kg/d in 2 divided doses) or levofloxacin (10–20 mg/kg/d) were prescribed, considering age and potential side effects.^[22]^

The decision to initiate corticosteroids or change antibiotics was based on the following criteria: persistent high fever (≥38.5°C) for <7 days, elevated inflammatory markers such as CRP ≥ 44.45 mg/L, LDH ≥ 590 IU/L, FER ≥ 411 ng/mL, neutrophil percentage ≥73.75%, and radiographic evidence of pulmonary consolidation or pleural effusion.^[23]^

### 2.5. Statistical analysis

All the analyses were performed using R 3.6.3 (http://www.r-project.org) and EmpowerStats (www.empowerstats.net, X&Y Solutions Inc., Boston).^[24,25]^ Continuous variables were expressed as means with standard deviations or medians with interquartile ranges, as appropriate. Categorical variables were expressed as frequencies and percentages. The relationship between WBC count and RMPP was analyzed using linear regression models. Both unadjusted and adjusted models were developed, with the latter controlling for potential confounders such as gender, age, weight, and other inflammatory markers. A threshold effect analysis was performed using a 2-segment linear regression model to identify any critical WBC levels that significantly alter the relationship between WBC count and RMPP. Statistical significance was set at a *P*-value of <.05. We assessed model assumptions to ensure robustness. Linearity between WBC count and RMPP risk was examined, supporting the use of a 2-piecewise linear regression model. Multicollinearity among covariates was assessed using variance inflation factors, with all values within acceptable limits. Model fit was evaluated using the Akaike Information Criterion and log-likelihood ratio test, confirming the superiority of the threshold model.

## 3. Results

We analyzed baseline demographic and clinical characteristics of pediatric MPP patients stratified by WBC tertiles. Significant differences existed across WBC groups. Higher WBC counts were associated with significantly higher inflammatory marker levels, including CRP, IL-6, and γ-IFN (all *P* < .001). Liver function parameters showed mild variations; alanine aminotransferase (ALT) slightly increased (*P* = .015), while aspartate aminotransferase (AST) exhibited a decreasing trend (*P* = .047). LDH and lactate levels significantly increased in the high WBC tertile (*P* = .044 and *P* < .001, respectively). Patients in the high WBC tertile were younger and had lower body weight (both *P* < .001). RMPP cases increased significantly across WBC tertiles (low: 25.99%, middle: 29.21%, high: 40.56%; *P* < .001; Table 1).

**Table 1:** Baseline demographic and clinical characteristics of pediatric patients with MPP stratified by WBC tertiles.

WBC tertile	Low	Middle	High	*P*-value
N	354	356	355	
Weight, kg	24.16 (9.15)	22.39 (8.44)	21.59 (9.22)	<.001
Age, yr	7.11 (2.55)	6.58 (2.55)	6.21 (2.82)	<.001
Gender				.295
Male, n (%)	178 (50.28%)	178 (50.00%)	196 (55.21%)	
Female, n (%)	176 (49.72%)	178 (50.00%)	159 (44.79%)	
N%	62.35 (54.30–70.27)	64.90 (58.13–71.35)	66.00 (57.35–74.50)	.002
CRP, mg/L	6.79 (2.48–17.68)	10.31 (4.50–18.15)	13.14 (6.03–22.19)	<.001
ESR, mm/h	24.81 (15.92–33.80)	27.41 (17.46–36.48)	24.11 (15.34–39.46)	.141
ALT, U/L	14.00 (11.00–19.00)	15.00 (11.00–20.00)	15.00 (12.00–21.00)	.015
AST, U/L	36.00 (28.00–48.00)	34.00 (28.75–47.25)	33.00 (25.00–47.00)	.047
LDH, U/L	338.00 (265.25–514.75)	327.00 (272.00–530.50)	369.00 (283.50–546.00)	.044
Lactate, mmol/L	1.70 (1.40–2.30)	1.75 (1.40–2.50)	2.10 (1.60–2.70)	<.001
IL-6, pg/mL	6.79 (2.48–17.68)	13.45 (6.30–26.10)	13.70 (6.70–28.40)	<.001
γ-IFN, pg/mL	3.20 (2.00–5.40)	4.60 (2.90–8.43)	5.90 (3.40–12.17)	<.001
RMPP, n (%)				<.001
No	262 (74.01%)	252 (70.79%)	211 (59.44%)	
Yes	92 (25.99%)	104 (29.21%)	144 (40.56%)	

Results in table: mean (SD)/median (Q_1_–Q_3_)/N (%).

CRP = C-reactive protein, ESR = erythrocyte, LDH = lactate dehydrogenase, N = neutrophil, RMPP = refractory *Mycoplasma pneumoniae* pneumonia, WBC = white blood cell.

WBC count was significantly associated with RMPP risk. Each 1 × 10⁹/L increase in WBC count corresponded to a 10% higher RMPP risk (unadjusted OR = 1.10, 95% CI: 1.06–1.14, *P* < .0001). This association persisted after adjusting for age, gender, and weight (adjusted OR = 1.10, 95% CI: 1.06–1.15, *P* < .0001) and remained significant after further adjusting for inflammatory and metabolic markers (OR = 1.10, 95% CI: 1.05–1.15, *P* < .0001). Patients in the highest WBC quartile (Q_4_) had a 2.35-fold greater risk of RMPP compared to the lowest quartile (Q_1_; OR = 2.35, 95% CI: 1.57–3.51, *P* < .0001), whereas Q_2_ and Q_3_ showed no significant risk increase. Analyzing WBC quartiles continuously revealed a 34% incremental risk per quartile (OR = 1.34, 95% CI: 1.18–1.52, *P* < .0001; Table 2).

**Table 2:** Association between WBC (×10^9^/L) and RMPP in pediatric patients, adjusted for various confounders.

Exposure	Non-adjusted	Adjust I	Adjust II
WBC × 10^9^/L	1.10 (1.06, 1.14) <.0001	1.10 (1.06, 1.15) <.0001	1.10 (1.05, 1.15) <.0001
WBC × 10^9^/L quartile			
Q_1_	1.0	1.0	1.0
Q_2_	1.01 (0.68, 1.48) .9793	1.02 (0.69, 1.50) .9264	1.03 (0.68, 1.55) .8913
Q_3_	1.24 (0.85, 1.81) .2614	1.26 (0.86, 1.84) .2362	1.30 (0.87, 1.94) .2076
Q_4_	2.22 (1.54, 3.19) <.0001	2.26 (1.56, 3.27) <.0001	2.35 (1.57, 3.51) <.0001
WBC × 10^9^/L quartile continuous	1.31 (1.17, 1.47) <.0001	1.32 (1.17, 1.49) <.0001	1.34 (1.18, 1.52) <.0001

Table data: OR (95% CI) *P*-value.

Outcome variable: RMPP.

Exposure variables: WBC; WBC quartile; WBC quartile continuous.

Non-adjusted model adjust for: none.

Adjust I model adjust for: age; gender; weight.

Adjust II model adjust for: age; gender; weight; N; CRP; ESR; ALT; AST; LDH; lactate; IL_6; γ-IFN.

CI = confidence interval, CRP = C-reactive protein, ESR = erythrocyte, LDH = lactate dehydrogenase, OR = odds ratio, N = neutrophil, RMPP = refractory *Mycoplasma pneumoniae* pneumonia, WBC = white blood cell.

**Figure 2.**
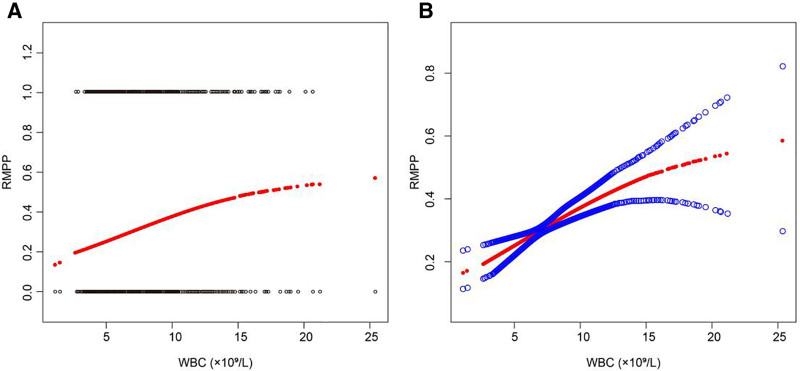
Threshold effect of white blood cell (WBC) count on the risk of refractory *Mycoplasma pneumoniae* pneumonia (RMPP). This figure presents a threshold effect analysis illustrating the nonlinear relationship between WBC count (×10⁹/L) and the probability of developing RMPP. The left scatter plot (A) depicts individual data points (red and blue dots), highlighting the distribution of RMPP cases at varying WBC levels. The right graph (B) shows fitted regression curves that demonstrate the risk trend, with a threshold (*K* = 14.3 × 10⁹/L) identified, marking a significant shift in the relationship. RMPP = refractory *Mycoplasma pneumoniae* pneumonia, WBC = white blood cell.

A critical threshold of 14.3 × 10⁹/L in WBC count emerged as a strong predictor of RMPP development. Nonlinear threshold analysis demonstrated that WBC levels exceeding this point serve as a robust clinical indicator of potential RMPP progression. While increases in WBC below this threshold showed a significant 20% risk increment per 1 × 10⁹/L (odds ratio [OR] = 1.2, 95% CI: 1.1–1.2, *P* < .001), WBC elevations beyond 14.3 × 10⁹/L no longer correlated with increased risk (OR = 0.9, 95% CI: 0.7–1.1, *P* = .231; Table 3). This finding underscores the pivotal role of 14.3 × 10⁹/L as a critical diagnostic marker, strongly suggesting clinicians should intensify monitoring and consider more aggressive interventions when patients’ WBC counts surpass this threshold (Fig. 2).

**Table 3:** Threshold effect analysis for exposure: WBC.

Outcome	RMPP
Model I	
Linear effect	1.1 (1.1, 1.2) <.001
Model II	
Threshold (*K*)	14.3
Effect segment 1 (<*K*)	1.2 (1.1, 1.2) <.001
Effect segment 2 (>*K*)	0.9 (0.7, 1.1) .231
Log-likelihood ratio test	0.017

Data in table: OR (95% CI) *P* value (first, check the *P* value in the last row (log-likelihood ratio test). If the *P* value is significant (<.05), choose the results from model II. If the *P* value is not significant, opt for the results from model I).

Outcome variable: RMPP.

Exposure variable: WBC × 10^9^/L.

Adjusted variables: gender; weight; N; CRP; ESR; ALT; AST; LDH; lactate; IL_6; γ-IFN; age.

CI = confidence interval, CRP = C-reactive protein, ESR = erythrocyte, LDH = lactate dehydrogenase, OR = odds ratio, N = neutrophil, RMPP = refractory *Mycoplasma pneumoniae* pneumonia, WBC = white blood cell.

**Figure 3.**
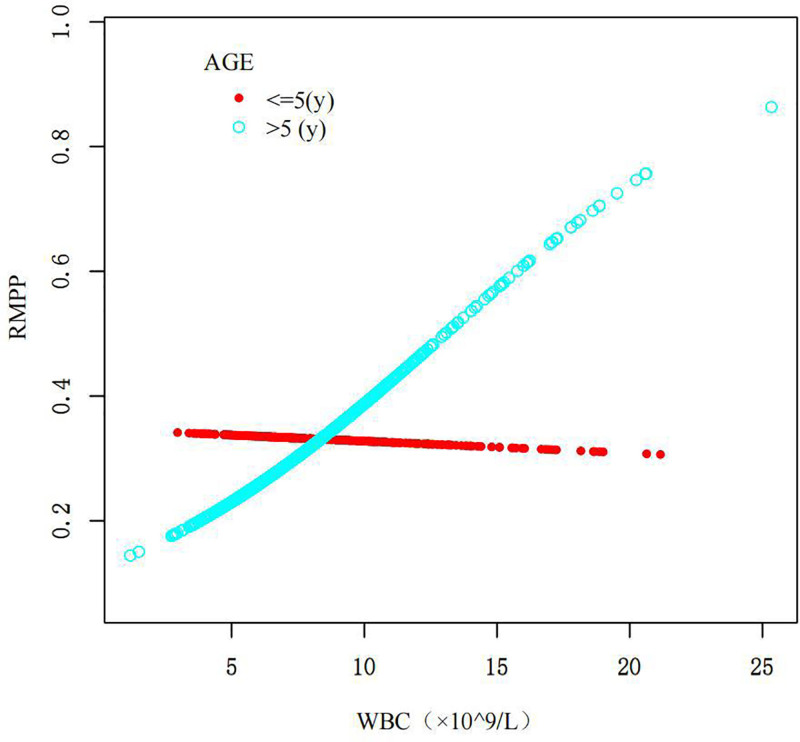
Relationship between white blood cell (WBC) count and the risk of RMPP. This figure illustrates the relationship between WBC count (×10⁹/L) and the probability of developing RMPP. The scatter plot displays individual data points, with red circles representing patients aged ≤5 yr and light blue circles representing those aged >5 yr. The varying patterns indicate different trends in RMPP risk associated with WBC levels across age groups. RMPP = refractory *Mycoplasma pneumoniae* pneumonia, WBC = white blood cell.

Interestingly, we performed a stratified analysis by age to evaluate how WBC affects the incidence of RMPP.^[26,27]^ After examining the *P*-value from the log-likelihood ratio test, which was not significant, we decided to rely on the results from model I (Table 4). In the ≤5 years group, the OR was 0.9 (95% CI: 0.8–1.0) with a *P*-value of .055, indicating no significant difference. Conversely, in the >5 years group, the OR was 1.2 (95% CI: 1.1–1.3) with a *P*-value of <.001, highlighting a significant association between higher WBC levels and an increased risk of RMPP (Fig. 3).

**Table 4:** Stratified analysis of the effect of WBC on RMPP by age group.

Outcome:	RMPP	
Age, yr	≤5	>5
Model I		
Linear effect	0.9 (0.8, 1.0) .055	1.2 (1.1, 1.3) <.001
Model II		
Threshold (*K*)	15.6	13.4
Effect segment 1 (<*K*)	1.0 (0.9, 1.1) .479	1.3 (1.2, 1.3) <.001
Effect segment 2 (>*K*)	0.5 (0.2, 1.1) .083	1.0 (0.8, 1.2) .945
Log-likelihood ratio test	0.072	0.062

Data in table: OR (95% CI) *P* value (first, check the *P* value in the last row (log-likelihood ratio test). If the *P* value is significant (<.05), choose the results from model II. If the *P* value is not significant, opt for the results from model I).

Outcome variable: RMPP.

Exposure variable: WBC × 10^9^/L.

Adjusted variables: gender; weight; N; CRP; ESR; ALT; AST; LDH; lactate; IL_6; γ-IFN; age.

CI = confidence interval, CRP = C-reactive protein, ESR = erythrocyte, LDH = lactate dehydrogenase, OR = odds ratio, N = neutrophil, RMPP = refractory *Mycoplasma pneumoniae* pneumonia, WBC = white blood cell.

## 4. Discussion

The present study investigated the threshold effect of WBC count on the risk of RMPP in pediatric patients, revealing a nonlinear association. The log-likelihood ratio test confirmed that a threshold model (model II) better explained the data than a simple linear model. A critical WBC threshold of 14.3 × 10⁹/L was identified, with each 1 × 10⁹/L increase below this threshold associated with a 20% higher risk of RMPP (OR = 1.2, 95% CI: 1.1–1.2, *P* < .001). However, when WBC levels exceed 14.3 × 10⁹/L, the association with RMPP risk becomes nonsignificant (OR = 0.9, 95% CI: 0.7–1.1, *P* = .231), indicating a critical threshold that strongly suggests the development of RMPP. This plateau effect implies that beyond this threshold, rather than experiencing continuous inflammatory escalation, patients may face a state of immune dysregulation. These findings underscore the importance of WBC count as a key biomarker for RMPP risk stratification. Moderate elevations in WBC count signal the need for early clinical intervention, while extreme elevations warrant a thorough evaluation of immune function, moving away from the assumption that increased WBC always indicates worsening inflammation.

Our findings align with previous research highlighting immune dysfunction as a key factor in RMPP pathogenesis. It has been well-documented that excessive host immune responses, rather than uncontrolled bacterial proliferation, drive disease severity in RMPP.^[1,7]^ Inflammatory markers such as CRP, IL-6, and γ-IFN have been implicated in disease progression, with studies suggesting that elevated IL-6 and LDH levels are strong predictors of RMPP severity.^[18]^ The observed plateau effect of WBC count beyond 14.3 × 10⁹/L suggests that excessive leukocytosis may not reflect an ongoing inflammatory state but rather immune exhaustion, where further increases in WBC fail to contribute to disease progression. This finding aligns with recent studies emphasizing the role of immune regulation in severe pediatric pneumonia, where excessive inflammation may transition into immune suppression, leading to ineffective bacterial clearance.^[11,17]^

Furthermore, our study provides critical insights into the role of WBC as a predictive biomarker. While WBC count is widely used in clinical practice, its predictive value for RMPP has been debated.^[17]^ Some studies have suggested that WBC count alone is insufficient to distinguish RMPP from general MPP, emphasizing other inflammatory markers such as neutrophil-to-lymphocyte ratio and CRP.^[17,18]^ However, our study demonstrates that a specific WBC threshold (14.3 × 10⁹/L) may enhance predictive accuracy, providing an objective cutoff for risk stratification. The threshold-dependent risk pattern observed in our study aligns with prior work indicating that moderate elevations in inflammatory markers often correlate with disease severity, whereas extreme elevations may signal immune dysregulation rather than active infection.^[7]^ This further highlights the complex immune mechanisms underlying RMPP, where WBC count alone cannot fully capture disease dynamics without considering other inflammatory pathways.^[12]^

The clinical implications of our findings are significant. Early recognition of RMPP is essential for preventing complications such as necrotizing pneumonia, acute respiratory distress syndrome, and multi-organ involvement.^[11,12]^ Current guidelines recommend early intervention with corticosteroids or second-line antibiotics (tetracyclines, fluoroquinolones) in suspected RMPP cases, but the criteria for early risk stratification remain unclear.^[7]^ Our results suggest that patients with WBC counts approaching 14.3 × 10⁹/L should be closely monitored, as they are at high risk of developing RMPP. However, clinicians should be cautious in interpreting extreme WBC elevations (>14.3 × 10⁹/L) as a sign of worsening disease, as this may reflect an immune regulatory shift rather than an ongoing inflammatory process. This highlights the need for comprehensive immune profiling, incorporating other inflammatory markers (IL-6, γ-IFN) and immune cell subpopulation analysis to guide treatment decisions.^[17,18]^ In addition, stratified analysis revealed that the association between WBC count and RMPP risk was significant only in children older than 5 years, suggesting potential age-related differences in host immune response.

This may be attributed to immunological maturation, as older children have a more developed adaptive immune system that may contribute to exaggerated inflammatory responses upon *Mycoplasma pneumoniae* infection. In contrast, younger children tend to rely more on innate immunity and may exhibit a more regulated leukocyte response.

Furthermore, school-aged children may have greater community exposure and higher rates of macrolide-resistant *M pneumoniae*, resulting in delayed diagnosis and more pronounced systemic inflammation. These findings indicate that WBC-based risk stratification may be particularly applicable in children over 5 years of age, and future research should explore age-specific cutoff values.

Despite the strengths of our study, several limitations must be acknowledged. First, this study was conducted at a single tertiary care center, which may limit the generalizability of our findings to other clinical settings or regions with differing epidemiology or treatment practices. Multicenter prospective studies are essential to validate the identified WBC threshold across diverse populations and healthcare systems. Such studies would also enable exploration of geographic and demographic variability in immune response and pathogen resistance patterns. Second, while WBC count was identified as a key biomarker, other immune parameters such as cytokine levels, lymphocyte subsets, and genetic predispositions were not analyzed, which could provide further mechanistic insights. Third, treatment protocols, including the timing and dosage of corticosteroids and the selection of antibiotics, were not standardized across all patients and may have influenced clinical outcomes. This variability represents a potential confounding factor that could affect the relationship between WBC count and RMPP progression. Although corticosteroid initiation was guided by defined clinical criteria, further prospective studies with uniform treatment protocols are needed to eliminate this source of variability. Future studies should incorporate comprehensive immune profiling and standardized treatment protocols to further elucidate the role of WBC count in RMPP pathogenesis and prognosis.

## 5. Conclusions

This study underscores the clinical significance of WBC count as a potential biomarker for risk stratification in pediatric RMPP. The identification of a threshold effect suggests that WBC elevations should not be interpreted in a linear manner but rather within the broader context of immune response dynamics. These findings highlight the need for more precise diagnostic criteria and tailored management strategies to improve early recognition and treatment of RMPP. Future research should focus on validating WBC thresholds across diverse populations, incorporating immune profiling to better understand the role of inflammation and immune dysregulation in disease progression. Additionally, the integration of machine learning models using multi-biomarker datasets could enhance risk prediction and individualized treatment approaches.

## Acknowledgments

We thank Yana Wang for her valuable assistance in data collection.

## Author contributions

**Data curation:** Xuan Wenjie, Yana Wang.

**Writing – original draft:** Lingke Liu.

**Writing – review & editing:** Xiaoxian Wang.
